# Negotiated learner modelling to maintain today’s learner models

**DOI:** 10.1186/s41039-016-0035-3

**Published:** 2016-04-23

**Authors:** Susan Bull

**Affiliations:** Institute of Education, University College London, London, UK

**Keywords:** Negotiated learner models, Open learner models, Visualisation, Intelligent tutoring systems, Multiple learning resources

## Abstract

Today’s technology-enabled learning environments are becoming quite different from those of a few years ago, with the increased processing power as well as a wider range of educational tools. This situation produces more data, which can be fed back into the learning process. Open learner models have already been investigated as tools to promote metacognitive activities, in addition to their potential for maintaining the accuracy of learner models by allowing users to interact directly with them, providing further data for the learner model. This paper suggests the use of negotiated open learner models as a means to both maintain the accuracy of learner models comprising multiple sources of data and prompt learner reflection during this model discussion process.

## Introduction

The technology-enabled learning contexts of today have become very different from those of even quite recent years. There are many more tools; there is much greater processing power; and perhaps, therefore, there is an even greater need for learner modelling to not only keep track of all this information but also to visualise learning data to make it meaningful to users. The next section introduces examples of intelligent tutoring systems as a basis to understand learner modelling research. The section that follows provides some examples of current directions of learner modelling, and the final section of the introduction presents the visualisation of learning data, ending with the current need for maintaining the accuracy of learner models comprising data from multiple sources.

### Intelligent tutoring systems

Traditional intelligent tutoring systems have modelled the individual learner’s skills, knowledge or strength of knowledge in a domain, and sometimes also their specific difficulties and/or misconceptions, and even attributes such as their motivation, goals and learning style (see, e.g. Woolf 2009). Using this *learner model*, which is inferred during the learner’s use of the system (based on, perhaps, their answers to questions, problem-solving attempts, tasks or subtasks attempted or completed, etc.), the system is able to appropriately personalise the interaction to the individual learner according to their current educational needs. This personalisation that is based on the learner model may result in the system offering a range of interventions or interaction types, which may include the following: additional exercises or tasks similar to those already undertaken, for consolidation of knowledge; explanations of how to proceed, or where and how a response was off-track; tutoring on related topics or concepts; and suggestions for navigation.

A simple example of an intelligent tutoring system is Subtraction Master (Bull and McKay 2004), which was designed based on known problems that children have with subtraction (Brown and Burton 1978; Burton 1982). Subtraction Master offers one-, two- and three-column subtraction questions targeted at the learner’s level, according to their learner model. These questions require children to type in their responses, which are then matched against the domain rules. The learner model includes representations of both the user’s subtraction abilities and any misconceptions identified (according to the subtraction studies referred to above) and is an open learner model (OLM—a learner model that can be accessed in a user-understandable form). The system then provides explanations in a suitable form matching their ability to follow the explanation; changes the difficulty level of questions; or shows the learner model to the learner, as appropriate.

In contrast, Guru (Olney et al. 2012) was designed based on teaching approaches observed in expert human tutors. It has an overlay learner model on the domain concept map for a biology curriculum and an accompanying textbook. Using natural language understanding techniques, Guru analyses student utterances (e.g. typed answers, summaries, responses to cloze exercises, completion of concept maps) to obtain data for the learner model and then provides appropriate instruction and formative feedback. For example, the animated tutor may ask a student to complete a concept, may ask a question to probe their understanding or may request verification of knowledge. Dialogue cycles with feedback cover the concepts according to the learner’s needs.

These two examples illustrate the conventional structure of intelligent tutoring systems (which comprise domain, learner and pedagogical models), for both a small restricted domain and a larger curriculum, and use in-depth knowledge about typical learner misconceptions to model the learner and focus the interaction (Bull & McKay, 2004), or knowledge of tutoring as performed by expert human tutors to guide the interaction (Olney et al., 2012). Many other approaches to designing and building intelligent tutoring systems also exist (see Woolf 2009), and impressive learning gains can be achieved (e.g. Corbett 2001).

### Some current directions of learner modelling research

It is now being recognised that the field of artificial intelligence in education, where intelligent tutoring systems have been developed, has broadened to include rich collections of digital materials (Kay 2012), and a main technical challenge for intelligent tutoring systems today relates to the speed with which information and communication technologies are developing (Cerri et al. 2012): this unavoidably influences users’ approaches to learning and their expectations about learning. Furthermore, people now use multiple applications and devices and obtain information in new ways. Recent advances in learner modelling approaches aim to encompass these, for example:

- The development of learning environments that are designed to hold diverse data from different sources in the learner model (Bull et al. 2012; Mazzola and Mazza 2010; Morales et al. 2009)
- A generic approach to integrate and edit learner models that are drawn from different learning resources (Cruces et al. 2010)
- Learner modelling across multiple applications where data must be transferred between them, combining e-portfolios and viewable learner models (Raybourn and Regan 2011)
- The introduction of a framework for exchanging learner profiles between sources, which includes the evidence for the data, enabling a different system to be able to appropriately interpret the meaning of the data (Dolog and Schaefer 2005)
- OLMs as a useful approach to help learners monitor their progress and plan their learning in MOOCs (Kay et al. 2013);
- OLMs to support self-regulation in blended learning using a learning management system (Tongchai 2016)

Independent open learner models are OLMs that are not embedded in a teaching system (Bull and Kay 2010), and, while they have historically been quite small scale, they can also be applied in the new learning contexts of today. This takes the learner modelling out of the traditional intelligent tutoring system context, such as exemplified above by Subtraction Master (Bull and McKay 2004) and Guru (Olney et al. 2012), to provide a focus for reflection and other metacognitive behaviours and give greater control over decisions in learning to the learner. Small-scale examples include Flexi-OLM (Mabbott and Bull 2004), which models knowledge and misconceptions in C programming based on multiple choice questions and short programming excerpts, and OLMlets (Bull et al. 2010), which models knowledge and misconceptions based on teacher-provided multiple choice questions on any subject. On a larger scale, and in line with the multiple data source learner modelling approaches described above, the domain-independent Next-TELL OLM accepts manual assessments from students and teachers, as well as automated data from external systems through its application program interface (API), and visualises these to learners and teachers in an aggregated form, or broken down by data source (Bull et al. 2013a).

### Learning analytics and open learner model visualisations

In addition to following the new directions of learner modelling described above, this potentially large-scale approach is in line with the developing and currently popular field of learning analytics. Learning analytics aims to support pedagogical approaches by providing assistance, often to the teacher, on some of the practical issues they face in their teaching (e.g. the quality of learning material or engagement of students with specific exercises). This typically involves monitoring learners’ actions and interactions with tools, and perhaps with peers (e.g. Lockyer and Dawson 2011), and using learning analytics visualisations, course instructors may be helped to optimise their teaching strategies (Dychkoff et al. 2012). The potential for learning analytics ‘dashboards’ as a useful way to display learning data is being recognised (Brown 2012; Charleer et al. 2014; Duval 2011; Verbert et al. 2013). There is also some initial recognition of the potential for learning analytics dashboards for use by students, to facilitate reflection and planning (Corrin and de Barba 2014; Durall and Gros 2014) and to inform learners of their progress towards attaining the required competencies (Grann and Bushway 2014).

Table 1 shows the more typical use of classroom learning analytics and open learner model visualisations to date. While there are certain exceptions, learning analytics visualisations, with a focus on performance scores or behavioural or interaction data, are often designed for teachers. For example, teachers can see which materials are being used, how successful student groupings are in terms of relative contributions, or how well students perform on different quizzes, and they can then change resources or adapt their teaching accordingly. In contrast, OLMs provide information about understanding, skills, competencies, etc. As well as being useful for teachers to help them understand their students’ learning state and therefore their needs, this type of information is also meaningful for learners. It can help them to plan, monitor and reflect on their learning, as well as support them in their independent learning. Of course, learning analytics visualisations can also be useful for learners, as in the examples above. The difference is that to date, this has been a less common aim in learning analytics. The nature of OLMs is that they provide visualisations that reflect *current* competencies and are usually designed specifically for use by the learner, to help support their decision-making.

**Table 1:** The space of visual learning analytics and open learner models

Visualisation type	For teachers (and other professional stakeholders)	For students (and perhaps teachers, parents, peers)	Activity, behaviour, performance score data	Inferred learning information	Large scale	Small scale
Learning analytics	x		x		x	
Open learner models		x		x		x

Because their origins are in big data and educational data mining, learning analytics visualisations often show larger scale data than OLMs, which have their roots in intelligent tutoring systems. However, links are beginning to be made between learning analytics visualisations and OLMs (e.g. Bull et al. 2013b; Durall and Gros 2014; Ferguson 2012; Kalz 2014; Kay and Bull 2015), so we can anticipate future benefits from building on research in both areas. There are now new opportunities to be had from combining the larger scale learning analytics approaches and open learner models that visualise learning or understanding.

Aims of OLMs for students include the following: to overcome the privacy issue of holding electronic data about learners without them having access to it; to improve the accuracy of the learner model by allowing user input to correct it or help keep it up to date; to encourage reflection on the learning process by showing learners representations of their understanding; supporting planning of learning episodes by making the learner model explicit to the learner; and increasing learner trust in the system by allowing students to see the information upon which teaching decisions are based (Bull and Kay 2007). Furthermore, (independent) open learner models can result in significant learning gains and increased self-assessment accuracy (e.g. Brusilovsky et al. 2015; Kerly and Bull 2008; Long and Aleven 2013; Mitrovic and Martin 2007; Shahrour and Bull 2009).

This paper provides guidelines for maintaining the accuracy of overarching OLMs that take data from multiple sources as in the examples above, by allowing learner and teacher input to the diagnostic and learner modelling process. This is important because a learner model that is constructed from different sources of data may more easily result in a quickly outdated, or an inaccurate, model: different systems and activities may produce performance data of differing validity, and some learning data may be more detailed, while other learning data may not be transferable to the overall OLM. This process of maintaining the learner model, in addition to offering a different task to increase motivation (Thomson and Mitrovic 2010), can also be a strong facilitator of metacognitive activities such as reflection and planning (Bull and Kay 2013). This approach therefore encompasses both the (potentially) big data of learning analytics and the new directions of intelligent tutoring systems and OLM research—providing a means to maintain the accuracy of learner models in today’s learning environments, while also continuing to provide the learning, metacognitive and motivational benefits of more traditional OLMs.

## Learner model visualisation

**Fig. 1:**
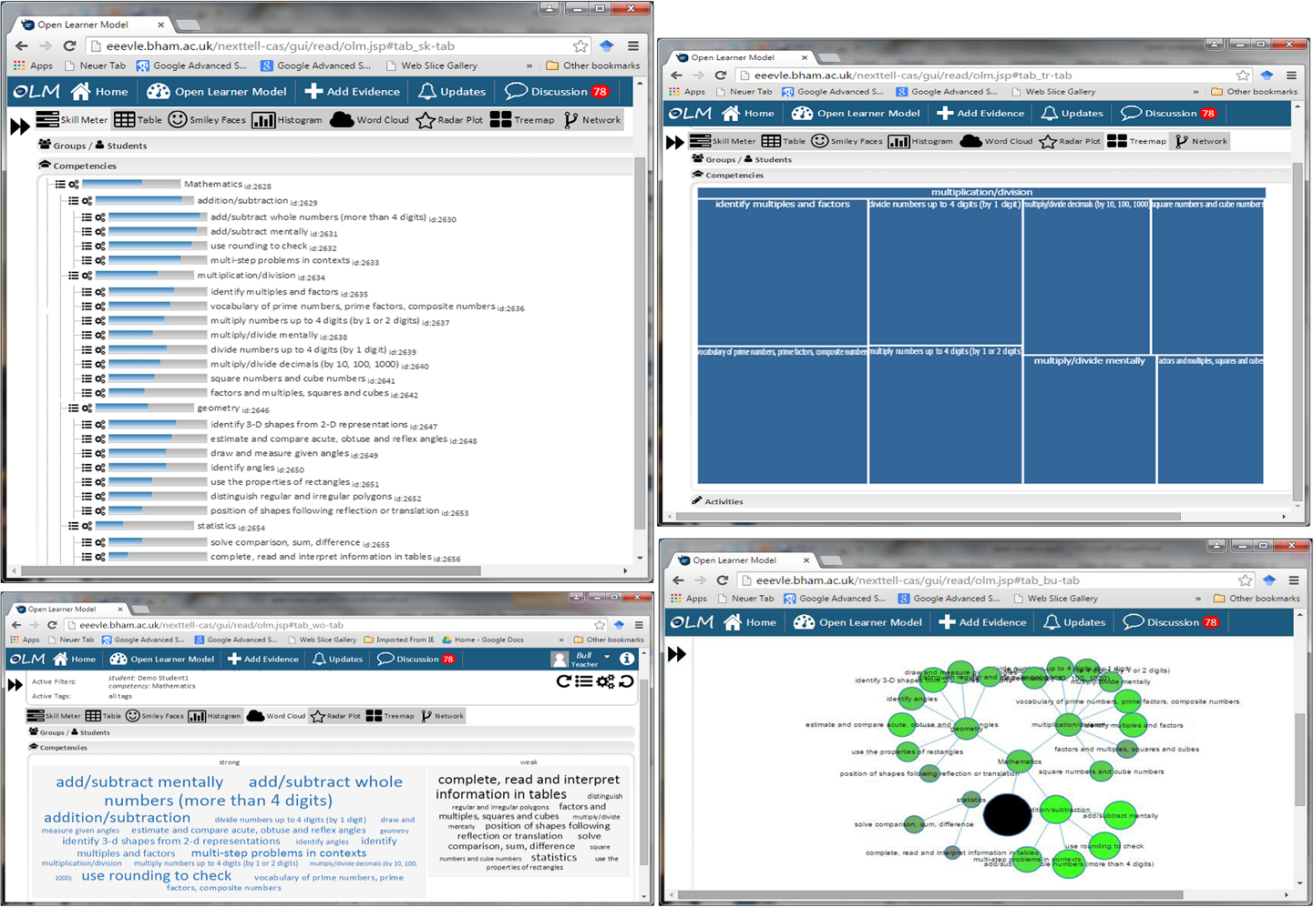
Four of the Next-TELL OLM visualisations

Various OLM visualisations have been used. The most common early visualisation, which is still used today, was skill meters (e.g. Bull et al. 2010; Corbett and Bhatnagar 1997; Long and Aleven 2013; Mitrovic and Martin 2007; Papanikolaou et al 2003; Weber and Brusilovsky 2001). There have also been examples of concept maps (e.g. Mabbott and Bull 2004; Perez-Marin et al. 2007) and hierarchical tree structures (e.g. Kay 1997; Mabbott and Bull 2004), amongst others. More recently, tree map overview-zoom-filter approaches to open learner modelling began to emerge (Bakalov et al. 2011; Bull et al. 2016; Kump et al. 2012), as well as tag/word clouds (Bull et al. 2016; Mathews et al. 2012) and sunburst views (Conejo et al. 2012). While many OLMs allow the user to access the model using a single visualisation, some systems display the learner model data using more than one visualisation (e.g. Bull et al. 2010; Conejo et al. 2012; Duan et al 2010; Johnson et al. 2013b; Mabbott and Bull 2004; Mazzola and Mazza 2010). This can be helpful given that users may have different preferences for how to access their learner model (Mabbott and Bull 2004; Sek et al. 2014). Figure 1 gives examples from the Next-TELL OLM, of hierarchical skill meters, tree map, word cloud and competency network (Bull et al. 2016; Johnson et al. 2013b). This example shows mathematics competencies, with the corresponding labels next to the skill meters, on the nodes of the competency network, in the word clouds and in the cells of the tree map (e.g. for multiplication/division, the sub-competencies are as follows: identify multiples and factors; vocabulary of prime numbers, prime factors, composite numbers; multiply numbers up to 4 digits (by 1 or 2 digits); multiply/divide mentally; divide numbers up to 4 digits (by 1 digit); multiply/divide decimals (by 10, 100, 1000); square numbers and cube numbers; factors and multiples, squares and cubes).

We here use the Next-TELL OLM as an example because it provides multiple views, which can easily illustrate the relative utility of each, in different situations. The hierarchical skill meters combine two of the more common approaches to opening the learner model visually, showing the structure of the corresponding domain in a linear fashion. While this is an advantage in terms of seeing the breakdown of information by topic, it is less useful for direct comparison of the extent of a learner’s understanding or competency, than single-level skill meters (e.g. Bull et al. 2010), because of the positioning of the skill meters relative to each other. This is a trade-off that needs to be considered in the design for each case of OLM use. The competency network shows the same structural information that, although less easily distinguished, gives a quicker overall impression by brightness and size of nodes and does not require scrolling to view many skills or competencies. The tree map is also useful in a smaller space, allowing users to zoom into sub-topics instead of seeing them all together, recognising the relative strength of skills by the size of the corresponding representation. The word cloud is more useful for a quick impression of extremes—it shows the strongest skills in larger (blue) text on the left and weaker skills in larger (black) text on the right. However, it is less helpful for identifying those skills closer to the borderlines of strong and weak. The Next-TELL OLM includes eight visualisations, more or less useful for different purposes (see Bull et al. 2016, for further detail).

In addition to visualising the contents of the learner model, some systems also make the reasoning or evidence for the learner model data available to the user. For example, in addition to the visualisations shown in Fig. 1, the Next-TELL OLM provides a list of each piece of evidence and its source (teacher, student, peer or automated assessment), including the weighting of the evidence, to allow users to gauge the extent and type of information contributing to the learner model data (Johnson et al. 2013a). Similarly, SIV displays a list of evidence alongside its OLM visualisation, for example, lecture slides viewed together with duration or tutorials completed (Kay and Lum 2005). In xOLM (Van Labeke et al. 2007), claims can be expanded to reveal nodes describing the corresponding evidence, as part of an approach to discussion of the learner model. In Prolog-Tutor, learning traces are available for consultation, to help learners decide whether to challenge the data (Tchetagni et al. 2007). In a contrasting approach, Czarkowski et al. (2005) highlight hypertext explanations that were included (in yellow) and excluded (in green) according to the learner model, for example: ‘This was included because your level was: basic’. This can help learners to decide whether the learner model is accurate and, consequently, whether they wish to change it.

The next section introduces learner models that allow users to influence the content of the learner model, before considering approaches to negotiating learner models composed of data from multiple sources.

## Negotiated and other types of interactive learner modelling

Given the possible size and complexity of learning data that may be in an individual’s learner model, gathered from multiple online activities and systems, some additional way of updating this model beyond the usual system inference based on student input during learning tasks is useful and, indeed, may be essential. This is particularly the case if some of the learning activities do not or cannot send data to the OLM. If these are the most recent activities, the OLM may quickly become outdated. This section first introduces some negotiated learner models and other systems that allow discussion of the learner model contents. Evaluation results are reported, before considering specific approaches that have been used to negotiate or influence the learner model.

### Systems that allow negotiation or discussion of the learner model

Some systems allow users to directly edit and therefore control the contents of the learner model (e.g. Bull et al. 2008; Czarkowski et al. 2005; Mabbott and Bull 2006); some allow learners to provide further information to try to persuade the system to change the information in their learner model such as by answering a short set of additional questions (e.g. Bull et al. 2007; Mabbott and Bull 2006; Tchetagni et al. 2007; Thomson and Mitrovic 2010), with the system ultimately retaining control, while yet others permit users to provide additional information that can be integrated into the learner model (e.g. Bull et al. 2013a; Kay 1997). *Negotiated learner models* allow the content of the learner model to be *discussed* and potentially updated according to the discussion outcomes. The aim of the interactive nature of this type of learner model should be an *agreed model*.

Mr Collins (Bull and Pain 1995) aims to increase the accuracy of the learner model via student-system negotiation of the model contents, as well as promoting learner reflection through this discussion process. The model is displayed in table form, with details in text. It holds two sets of belief measures: the system’s inferences about the student’s understanding and the student’s own confidence in their knowledge, with identical interaction moves available to the learner and system to resolve any discrepancies between their respective beliefs about the learner’s knowledge: e.g. the learner or system may challenge, offer evidence, request explanation, agree, disagree, suggest or accept compromise. This approach is based on Baker’s (1990) negotiated tutoring and notion of ‘interaction symmetry’. If discussion cannot resolve disagreement about the model data, both belief sets are retained in the learner model separately. During inspection and negotiation of the learner model, both sets of beliefs are shown to the learner.

Subsequent work extended the methods of negotiation in negotiated learner models from the original menu-based discussion (Bull and Pain 1995) to graphical representations of conceptual graphs, with learner model maintenance occurring through more sophisticated linguistic and philosophical-based dialogue games in STyLE-OLM (Dimitrova 2003). STyLE-OLM uses dialogue moves such as inform, inquire, challenge, disagree, justify, agree, suggest and skip (based on Pilkington’s (1999) scheme). A second extension of the original approach to negotiating the learner model is CALMsystem’s negotiation of a learner model displayed as smiley faces (with differing levels of ‘smiliness’) for children, with the same negotiation moves as Mr Collins’, but with negotiation occurring through a chatbot (Kerly and Bull 2008). Results of a Wizard-of-Oz study are being examined to determine the rules that will be required for a learner model negotiation dialogue based on behavioural and affective states in negotiation-driven learning (Suleman et al. 2015). It is envisaged that statements such as confidence, interest, confusion, evaluation and motivation will be required. While there are some differences between the terminology of the interaction or negotiation moves in the above systems, it can be seen that the general negotiation issues and moves are similar.

Similar to negotiated learner modelling is xOLM’s approach (Van Labeke et al. 2007), but in this case the student initiates the discussion rather than following the approach of interaction symmetry, where negotiation could be initiated by either the student or the system. For example, using a dynamic graphical representation, students can explore the learner model and challenge claims, warrants and backings (based on Toulmin’s (1959) argumentation model that uses data, claims, warrants, rebuttals and backings) and receive justifications from the system. The interaction with the graphical interface is transcribed as a persisting (readable) dialogue. Learners can choose to agree, disagree or move on (without resolution). Unlike the interaction symmetry of the full negotiation found in the previous systems, xOLM allows the learner’s challenge to succeed in cases of unresolved disagreement.

Similarly to xOLM, the discussion component of EER-Tutor’s learner model (Thomson & Mitrovic 2010) allows the learner to initiate dialogue with the system, in the form of a challenge to the learner model representations. Learners can view the skill meters and click on a concept to start a discussion aimed at persuading the system of their viewpoint. Unlike xOLM, the system retains control of whether the model is changed, according to responses to additional questions that the learner must answer on the concept. When viewing the learner model, both learner and system beliefs for the learner model are combined.

In contrast to the above examples, EI-OSM, an OLM for students, teachers and parents, defers the overriding decision to the (human) teacher in cases where student-teacher interaction cannot resolve discrepancies (Zapata-Rivera et al. 2007). EI-OSM is an evidence-based model that uses a simplified version of Toulmin’s (1959) argument model. Users can view the learner model as a proficiency map, which uses colour to indicate proficiency level. The evidence-based argument structure is revealed by clicking on proficiency nodes, and new evidence can be added by clicking on a ‘supporting evidence’ tab. Users can describe new evidence or select from a teacher-defined list.

CoNeTo (Vatrapu et al. 2012) is a graphical tool to offer a socio-cultural approach to negotiating about the learner model, which allows learners and teachers to discuss the Next-TELL OLM (Bull et al. 2013a), and teachers (or students) can then provide further data for the OLM to add evidence arising from the discussion—i.e. teachers manually provide the additional evidence based on the learner-teacher agreed learner competencies, including any learning that has occurred during the discussion (Bull & Vatrapu 2012).

Other approaches, while further from the negotiated learner model paradigm, are also of interest. For example, a Facebook group allowing university students to discuss their learner models with each other indicates the willingness of students to critically consider representations of their understanding in an open-ended way (Alotaibi and Bull 2012); children providing their own assessments of their knowledge to a system if they disagree with it, quantitatively and explained in text comments for viewing by the teacher, may become a focus for subsequent (human) teacher-child discussion of understanding (Zapata-Rivera and Greer 2004); and comparing one’s performance and contribution to a learning community to that of a selected peer’s profile may lead to online messages between peers (Shi and Cristea 2014).

In addition to the examples above that can permit other users to view and interact about an individual’s learner model, several OLMs have allowed learners to view each other’s learner models (e.g. Bull and Britland 2007; Bull et al. 2007; Hsiao et al. 2013; Shi and Cristea 2014; Papanikolaou 2015). Such approaches are relevant as a starting point to considering negotiated learner modelling, since they have already examined the issue of visualising the learner model not only to the user the model represents but also to other parties. This visualisation is usually in the same form as viewed by the model ‘owner’, which has the benefits of familiarity and aiding comparison or discussion.

In addition to visualising the content of the learner model, for learner models to be negotiated, a way of showing the evidence or reasoning is required. This is followed up in the “Visualisation” section. Furthermore, in our context of multiple sources of data and possible variation in quality or validity of data, it may be more likely that there will be uncertainty in the learner model representations. This uncertainty may also be shown to users by adapting the visualisation in some way (Demmans Epp and Bull 2015) or provided through showing the learner model evidence calculations (Bull et al. 2016; Johnson et al. 2013a).

In this paper, we are concerned with both supporting human-human discussion and automating this discussion as with previous negotiated learner models, while also encompassing a range of potential sources of learner model data—manual and automated—as in the Next-TELL OLM. Issues that may be important for negotiated learner models, based on requirements for ‘negotiated collaborative assessment’, include the following: assessment criteria and reasons for assessment criteria, extent of student challenge allowed with reference to assessment criteria, extent to which a learner can influence the negotiation outcome, additional relevant evidence, ground rules for negotiation and including learning during negotiation into the learner model (Brna et al. 1999). In our context of multiple data source OLMs, new issues arise, such as negotiating the weight of different sources of data according to their relative validity and the method of visualising the learner model that is appropriate to the source of data from which the learner model representation is being negotiated—e.g. is it a simple quiz score, or is there detailed associated reasoning?

### Evaluations of negotiated and other interactive learner models

In this section, we consider results of studies investigating the perceived utility of negotiated learner models or learning benefits demonstrated. Because learner model negotiation involves interactions such as challenging the model or (dis)agreeing with it, we include systems that do not have the interaction symmetry of fully negotiated learner models. In addition, while not negotiated models, some studies have suggested that allowing learners to directly change information in the learner model (where errors had been deliberately introduced by the system to determine whether students would correct their model) can lead to a more accurate learner model (Bull et al. 2008), and offering predictions of a learner’s expertise can motivate them to perform self-assessments for their learner model (Hochmeister et al. 2012). While the latter did not measure self-assessment accuracy, OLMs have indeed been found to increase the self-assessment of at least the weaker students at university level (Mitrovic and Martin 2007) and also of schoolchildren (Kerly and Bull 2008). This provides a positive starting point for negotiated learner models, since learners do appear able to recognise information they regard as an inaccurate representation of their understanding, as well as being able to propose updates. This is essential for negotiating a learner model.

Three approaches to true negotiated learner models (i.e. with identical interaction moves available) were identified above. In a study of Mr Collins (*n* = 9), positive attitudes were identified to negotiating the learner model using structured menus for negotiation moves (e.g. ‘system justify itself’, ‘accept system’s decision’, ‘accept compromise’, ‘justify myself’) and provision of evidence (e.g. ‘I have forgotten’, ‘I have read’) (Bull and Pain 1995). The learner model was displayed in table form, showing both student and system beliefs about the student’s knowledge for each type of sentence in a language-learning domain. Students did challenge the learner model when they disagreed with its contents. Furthermore, most were positive towards the system challenging them if it disagreed with their attempts to change their learner model.

A study of STyLE-OLM (*n* = 7) also found that learners were able to understand and build arguments, with the graphical conceptual graph representation of the domain of technical terminology helping learners to construct concepts and relationships (Dimitrova 2003). Learners believed that they would be able to influence the content of the learner model and appreciated the sentence-opener approach to discussion—for example, on clicking on ‘inform’, sentence-opener choices become available (e.g. ‘I think’ and ‘I don’t know’). It was suggested that more knowledgeable learners would engage in reflective interactions about the domain; less knowledgeable learners would be prompted to challenge their learner models.

Unlike the previous systems that were trialled in higher education settings, CALMsystem (Kerly and Bull 2008) was evaluated with 10–11-year-olds (*n* = 25, in inspectable or inspectable + negotiated condition). Viewing smilies as an indicator of level of understanding of various scientific concepts, students in the negotiated condition also engaged in dialogue with a chatbot. For example, they could freely give inputs such as: ‘why do we think differently?’, ‘what am I good at?’, ‘what’s your belief?’, ‘I have difficulty with [topic]’, ‘change my belief’, ‘what should I do next?’, ‘why do you think I have a low level for [topic]?’ All participants (both conditions) significantly reduced their mean error in self-assessment, with those in the negotiated condition showing a significantly greater improvement than those in the inspectable-only condition.

VisMod was evaluated with children aged 10–12 (*n* = 110), with discussion of the learner model occurring through text input (Zapata-Rivera and Greer 2004). The Bayesian model was presented showing separate nodes for student and system beliefs about the student’s understanding of cell biology, which were also combined in a single node. There was a tendency for students to increase their knowledge level rather than decrease it. Six groups were involved in the study: single student acting freely, single student following interaction protocol, single student with guiding agent, student pairs, student with teacher, and a group of five students. Where teachers and students worked with the OLM together, discussion led to concept-by-concept changes to the learner model, in line with the student-teacher dialogue. While students in the pair groupings would explain their learner models to peers, they were generally less interested in providing this information to the learner model. However, this was not the case in the student-agent group, where participants were happy to interact with the agent (which also encouraged them), and they provided explanations. The available self-assessment explanation options were also used (e.g. ‘I have done research’, ‘I have demonstrated it’). Students understood and were interested in interacting with their learner model to ensure that it was accurate.

Evaluation of EER-Tutor’s learner model discussion (*n* = 11) of knowledge of database design, for university students, offers positive learner perceptions of the approach of trying to persuade the system of the learner’s viewpoint (Thomson and Mitrovic 2010). Of particular interest is that it offered a different task, thereby increasing motivation. The overall highest-scoring item in the questionnaire was the ease of use of the interface, which comprises a set of skill meters that comfortably fits onto a screen and their corresponding concept labels. Challenging the learner model occurs straightforwardly by clicking on the concept about which the learner wishes to initiate interaction, whereupon an appropriate test item is given.

The other main systems considered in the previous section were xOLM (Van Labeke et al. 2007) and EI-OSM (Zapata-Rivera et al. 2007). In these cases, the discussion components were not part of the main study. We therefore present short summaries of the findings that may be of relevance to a negotiated learner model, albeit not with reference to negotiation or discussion. Evaluation of xOLM (*n* = 10), which has a complex method of interaction following the Toulmin (1959) argumentation patterns, was found to require some time for participants to gain sufficient familiarity. However, the Toulmin argument structure was then considered easy to use, and the topic map was found helpful in understanding the links between concepts (Van Labeke et al. 2007). EI-OSM was evaluated with teachers (*n* = 8), who found the proficiency map to be a useful representation for understanding strengths and weaknesses, especially the colour of nodes to represent proficiency level, the relationships displayed between the nodes and the possibility of being able to create instructional paths after seeing the learner models (Zapata-Rivera et al. 2007). Teachers also found the explicit assessment claims helpful and felt that they could use the OLM to decide on actions for individuals or for grouping students, as well as possibly changing classroom instruction. CoNeTo (Vatrapu et al. 2012), the tool designed specifically for learner-teacher discussion, has not yet been evaluated with target users.

While most of the studies described in this section were quite limited, there is sufficient evidence to suggest that properly designed, negotiated learner models or other methods of interactive learner modelling in today’s technology-rich learning contexts may be very valuable. The growing use of multiple devices and multiple activities and learning resources make it particularly important to have a mechanism to maintain accurate and current information about learning. While the new interest in learning analytics dashboards (e.g. Brown 2012; Charleer et al. 2014; Duval 2011; Verbert et al. 2013) go some way towards visualising activity data, OLMs naturally make this information more meaningful by displaying information about learning (as opposed to behaviour) (Bull et al. 2013b). Such meaningful representations allow negotiation of the learning data to be more easily used. Evidence or justification may be in the form of behaviours (as in most learning analytics visualisations), while it is the learner model that will be negotiated.

### Approaches to negotiating and discussing learner models

As observed above, some approaches to negotiating or interacting about the learner model are based on Toulmin’s (1959) argument pattern. These use:

- *Data* (facts, reasons or support for claim: e.g. a score for a question, a topic or a larger assessment)
- *Claims* (position, assertion or conclusion: e.g. ‘I think you are Level II’ (Van Labeke et al. 2007))
- *Warrants* (evidence, reasoning process—logical connections between data and claims: e.g. ‘if a person has low algebra proficiency, they will obtain low scores…’ (Zapata-Rivera et al. 2007))
- *Rebuttals* (exceptions or disagreements with claim, data or warrant: e.g. information that a visually impaired student received a specific test in the standard font size (Zapata-Rivera et al. 2007))
- *Backings* (information supporting warrant or rebuttal)

For example, in xOLM, Van Labeke et al. (2007) use each of the above in the learner model representation except rebuttal (the reason given is that the learner model evidence relates to abilities). As stated previously, discussion of the learner model is initiated by the learner. This takes the form of requests for explanations of judgements (e.g. ‘Why do you think I’m Level III at my ability to Solve Problems on Difference Quotient?’ (Van Labeke et al. 2007). This relates to a claim (see above). xOLM then provides a deeper explanation of the claim (or data) by expanding the tree style structure. The learner may question this further, which would lead to presentation of the first element of the warrant (evidence). This can be further expanded to display the backing (reasons). Learner challenges to warrants indicate learner disagreement with the use of specific evidence to form its claims (e.g. ‘I misunderstood the goal of the exercise’ (Van Labeke et al. (2007)). Challenges to backings show disagreement with the evidence (e.g. ‘I don’t think this exercise was so easy’ (Van Labeke et al. (2007)), and challenges to claims reflect disagreement with the way in which evidence is combined (e.g. ‘I understand your point, but…’ (Van Labeke et al. 2007)).

EI-OSM (Zapata-Rivera et al. 2007) integrates evidence about performance from a range of sources, and although not a negotiated learner model in the sense defined here (i.e. with identical interaction moves and identical powers), it is close to our context of multiple-source OLMs. In addition to test outcomes, EI-OSM allows students, teachers and parents to provide supporting evidence for assessment claims or give different explanations for them. The argumentation moves in EI-OSM are similar to those described above for xOLM, in that students can view the system’s claims about their knowledge and also the data and warrants. In addition, unlike xOLM, EI-OSM allows rebuttals to be viewed. The student may challenge the data, claim, warrant or rebuttal—for example, a rebuttal against a claim (e.g. <NAME> ‘actually has high ability’ (Zapata-Rivera et al. 2007)), the data (e.g. ‘the low score resulted from an incorrect key’ (Zapata-Rivera et al. 2007)) and the warrant (e.g. an argument is false).

We have already identified that not all learner model negotiation or discussion approaches use the above argument pattern but have seen that general dialogue moves are similar. For example, STyLE-OLM’s (Dimitrova 2003) dialogue moves—inform, inquire, challenge, justify, agree, disagree, suggest and skip—and Mr Collins’ (Bull and Pain 1995) and CALMsystem’s (Kerly and Bull 2008) discussion options—challenge, offer evidence, request explanation, agree, disagree, suggest or accept compromise—can be easily related to the approaches of xOLM (Van Labeke et al. 2007) and EI-OSM (Zapata-Rivera et al. 2007). Even the simpler forms of interacting with the learner model include some form of challenging or changing the model, for example where it can be directly edited (e.g. Bull et al. 2008; Mabbott and Bull 2006), or the student can attempt to persuade the system to change it by demonstrating their knowledge in a short quiz (e.g. Bull et al. 2007; Mabbott and Bull 2006; Tchetagni et al. 2007; Thomson and Mitrovic 2010). Systems that allow users to provide additional evidence for inclusion in the learner model also use some kind of action on the model (e.g. Bull et al. 2013a; Kay 1997). For the purpose of this paper, which is to consider learner model negotiation approaches for today’s context of multiple learning activities contributing data, the terminology adopted will be straightforward. It is difficult to predict the aims of individual systems or learning activities from which learner model data will come, and users may be at a range of levels—we have seen examples of discussion of learner models by children (Zapata-Rivera and Greer 2004) as well as children’s negotiation of the learner model content (Kerly and Bull 2008) and university students’ learner model discussion (Thomson and Mitrovic 2010) and negotiation (Bull and Pain 1995; Dimitrova 2003). In individual cases, designers may choose a certain argument or negotiation terminology over others.

## Negotiating learner models with multiple data sources: the next steps

In this section, we revisit the visualisation and externalisation of OLMs, and in the “Visualisation” section, we focus in particular on how negotiation can be recorded for ongoing or later examination. The negotiation or discussion phase is important to ensure an accurate learner model representation, taking into account issues such as the recency of evidence, weighting of evidence, and even absence of evidence (e.g. for tasks that do not pass data to the learner model). In addition to maintaining model accuracy, the negotiation of a learner model is in itself an important learning task: it can increase motivation (Thomson and Mitrovic 2010), it can lead to significant learning gains (Kerly and Bull 2008) and it can be a useful support and prompt for metacognitive behaviours such as raising learner awareness of issues that they need to (re)consider and greater understanding of their own learning processes (Bull and Kay 2013).

The “Terminology and negotiation moves” section follows with suggestions for general terminology that can be widely understood (i.e. by a range of users), though individual instances of negotiated learner models may find specific models and corresponding terminology more appropriate. For those readers, this section can be viewed more as a ‘template’. More generally, this description is intended as a basis for designing negotiated learner modelling approaches, which can be interpreted in a lightweight fashion or extended with greater detail, as appropriate. In all cases, trade-offs between model accuracy, usability, learner motivation and support for metacognitive processes will need to be considered. For example, although interacting about the learner model has been found to be motivating, as it is a change from the main learning task (Thomson and Mitrovic 2010), this was a short interaction where a learner challenge results in a test item being provided, so that the system can verify whether the learner’s claim about their knowledge is to be considered correct. With more in-depth discussion that explores the evidence for beliefs and offers a wider variety of interaction moves, accuracy of the learner model may be improved, but motivation may be affected differently.

Finally, in the “An illustration for negotiating learner models with multiple sources of data” section, we provide some possible examples for negotiating learner models built from multiple sources of data.

### Visualisation

There have been arguments supporting a variety of visualisation methods. Some may be designed for specific domains, such as graphical representations of the consequences of learner beliefs, that relate directly to representations of the domain (e.g. animations of learner beliefs in the same form as domain animations—demonstrated for programming steps and chemistry (Johan and Bull 2010)). Others may be more general, such as the findings described above that some map views or graphical representations that use nodes and links are considered helpful for identifying relationships (Van Labeke et al. 2007; Zapata-Rivera et al. 2007). However, sometimes choice of learner model visualisation may depend on learner preferences (Mabbott and Bull 2004; Sek et al. 2014). Therefore, our recommendations in this section will cover a range of learner model visualisations.

Another issue for consideration is that learner model representations may contain some uncertainty. It is uncommon for this uncertainty to be shown directly in OLM visualisations, though methods for doing so have been proposed, for example: changing the fill or opacity of skill meters; mapping opacity to emoticons (e.g. smilies) to show certainty; using three-dimensional displays to allow depth to relate to certainty; the arrangement (e.g. horizontal or multi-directional) of words in word clouds, with ‘untidy’ word clouds indicating greater uncertainty; changing grain or opacity of nodes to show uncertainty; and changing links (e.g. dashed lines or line width) according to certainty of user understanding of relationships between concepts (Demmans Epp and Bull 2015). Such uncertainty visualisation can be very helpful in negotiating learner models, and, while we do not address this explicitly here, we consider that such visualisations could further support learner understanding of differences between learner beliefs about their understanding and the system’s beliefs about the learner’s understanding.

In addition to learner model visualisations, we have also seen that the reasoning or the evidence for learner model data can be externalised to users (e.g. Bull and Pain 1995; Johnson et al. 2013a; Kay and Lum 2005 Tchetagni et al. 2007; Van Labeke et al. 2007; Zapata et al. 2007). In learner models that show both, as is necessary for negotiated learner modelling, attention needs to be given to how they can work together.

Returning to the Next-TELL OLM visualisation examples from Fig. 1, we see that some cases would often allow easy access to a negotiation component by simply clicking on the corresponding label. For example, the competency name next to a skill meter or in the word cloud. While retaining the visibility of the learner model data, a separate area can be opened up for interaction about the data. However, this method of initiating negotiation becomes more difficult with representations such as the competency network and the tree map, because clicking on these leads to the next layer of data becoming available: collapsing or expanding of nodes in the competency network and drill-down through the hierarchical structure in the tree map. It might be argued that some visualisations better lend themselves to ease of negotiation than others, but if we accept that a range of visualisations may be appropriate for the purpose of viewing the learner model, it is necessary to accommodate these. Most negotiated or other interactive learner modelling approaches have some kind of dual externalisation:

- Mr Collins (Bull and Pain 1995) maintains a table view of both parties’ beliefs, while allowing menu-based discussion that is recorded in text below the table.
  - e.g. System: You are trying to change the system’s confidence measure from 3 to 1. This new confidence value is too high. Your last five attempts to place the pronoun in positive main clause statements were the following: [attempts listed with errors indicated]. These recent attempts have demonstrated that you are still having some difficulties. You have probable transfer from Spanish for pronoun placement twice. You have omitted the hyphen once… What do you wish to do? (Options: accept system’s decision; system justify itself, accept compromise (2), justify myself…)
- STyLE-OLM (Dimitrova 2003) provides buttons that lead to sentence openers alongside the conceptual graph, with interaction recorded in text that is displayed under the conceptual graph visualisation.
  - e.g. System (inquire): do you think capital_market raises capital and capital_market operates with corporate_stock? Learner (disagree): I disagree capital_market operates with corporate_stock. System (inquire): do you think capital_market raises capital and capital_market operates with tax_exempt_bond?
- In CALMsystem (Kerly and Bull 2008), the smiley representations remain visible while the free text negotiation with the chatbot is recorded in a separate window alongside.
  - e.g. System: I believe that you have a high knowledge level for the evaporation of a solution topic. You have said that you have a low confidence level in your ability for this topic. We still need to resolve this difference. Would you like to: change your belief so that you agree with me? (The recommendation is high knowledge level.) OR see why I hold my views? (Have me explain.) OR view your and my beliefs about your knowledge? OR answer some questions to show me how much you know? (The student may type anything into the response box.)
- xOLM (Van Labeke et al. 2007) has a complicated mechanism for negotiating the learner model, with different views for different aspects of Toulmin’s (1959) argument pattern. A text record is displayed below the learner model visualisation.
  - e.g. System: Your ability to solve problems on difference quotient seems to be at level II. Student: I don’t understand what this means. System: This graph represents my judgement of your competency, on a continuous scale between level I and level IV. Being very close to the level II indicator, my conviction on my judgement is therefore quite strong. Student: Could you tell me why you think I am level II?
- Prolog-Tutor (Tchetagni et al. 2007) allows the learner to select a skill from a list, where coloured squares indicate the skill levels and evidence in the form of the dialogue of the previous session is shown to the right of the skill list.
  - e.g. System (challenge): Let’s analyze… Student (assertion): I agree with this. System (challenge): Justify your…
- In VisMod (Zapata-Rivera and Greer 2004), in contrast to the above examples, rather than viewing the nodes and relationships between nodes in the graphical representation of the Bayesian model, when learners use a slider to suggest changes to their model they see the various options for the node in question in the possible sizes and colours representing knowledge, but not in the context of the larger model. The interaction takes place through a dialogue box.
  - e.g. System: system/teacher opinion - you have basic knowledge of Nucleous (0.33). System explanation: incomplete definition - question 3. You think - I have a very good knowledge of Nucleolus (0.78). If you wish you can change your opinion. Explain why you think so. (Slider provided for numerical value, text box provided for explanation to be seen by (human) teacher, checkboxes provided for evidence: I am interested in this topic; I have done research (read, asked) about it; I have demonstrated it (tests, projects).)
- In EI-OSM (Zapata-Rivera et al. 2007), the evidence is listed in a separate window from the visualisation of the Bayesian model, to which students can add further evidence or alternative explanations in text, for the teacher.
  - e.g. System: Clarissa’s knowledge level of ‘calculate slope from points’ is low. The following tasks were incorrect. Task 12 [open] difficulty level = hard. Task 27 [open] difficulty level = easy.
- In the skill meters of EER-Tutor (Thomson and Mitrovic 2010), clicking on the concepts leads to a separate dialogue aimed at verifying the student’s claim through testing.
  - e.g. Student: I know more than you think I do about entities. System: Ok, when drawing EER diagrams, which sort of words in the problem text are likely to model entities?
- Using CoNeTo (Bull and Vatrapu 2012), students and teachers can discuss the learner model using a graphical tool, where links and relationships can be made between concepts, or the discussion can occur face to face without using the tool. Therefore, no direct record is automatically saved, but teachers and students can compose explanations to store in the learner model, if they wish.
  - e.g. Teacher: the weighting of the evidence is low compared to more extensive activities; the data has since been superseded; when aggregated with other data, this entry has relatively little influence.

The above suggests that, while learner model visualisations are often graphical, the discussion process may occur through graphical representations or a language-based dialogue. In some cases, both are an option. What does seem to be considered important is that there be a text record of the negotiation or discussion interaction, which can be considered at the time of discussion, or afterwards. Thus, for negotiating learner models, ‘visualisation’ in the form of text is relevant. Looking at the examples of these text-based argument records, it can be seen that these include the following: *statements of what the learner is trying to change in the learner model* (Bull and Pain 1995); *statements of beliefs* (Dimitrova 2003; Kerly and Bull 2008; Thomson and Mitrovic 2010; Van Labeke et al. 2007; Zapata-Rivera and Greer 2004; Zapata-Rivera et al. 2007); *enquiries for more information* (Dimitrova 2003; Thomson and Mitrovic 2010); *agreements* (Tchetagni et al. 2007); *disagreements* (Bull and Pain 1995; Dimitrova 2003; Kerly and Bull 2008); *statements of the evidence for a representation* (Bull and Pain 1995; Zapata-Rivera and Greer 2004); *explanations of the evidence for a representation* (Bull and Pain 1995; Bull and Vatrapu 2012; Zapata-Rivera and Greer 2004; Zapata-Rivera et al. 2007); *challenges* (Bull and Pain 1995; Kerly and Bull 2008; Tchetagni et al. 2007); and *explanation requests* (Tchetagni et al. 2007; Van Labeke et al. 2007; Zapata-Rivera and Greer 2004). Of course, these systems also include other dialogue moves—those given here are simply to illustrate the types of discussion found in excerpts from those discussions. Finally, while we have not explicitly considered models of collaborative learning skills (e.g. Soller et al. 1999) in this paper, the issues tend to overlap with the kinds of argument required in negotiated learner modelling.

### Terminology and negotiation moves

Building on the dialogue excerpts from the “Visualisation” section, the system descriptions in the “Approaches to negotiating and discussing learner models” section, and Brna et al.’s (1999) requirements for negotiated collaborative assessment in the “Systems that allow negotiation or discussion of the learner model” section, the main negotiation and discussion moves used or suggested, have been identified. However, the terminology used is not always the same.

The contexts of use considered in this paper are varied: learners may be at advanced higher education level or may be adults in other learning situations or they may be children; parents may have access to the learner models of their children, but their levels of education may be quite different from each other and, indeed, their children; and teachers, who can usually be expected to be highly educated, may have access to the learner models. Given this situation, as indicated above, we will adopt more general terminology that could be used in most cases, both to suit the individual user and to facilitate communication about the learner model between users, where this is relevant. Specific cases may choose to use different terminology (e.g. Van Labeke et al.’s (2007) and Zapata-Rivera et al.’s (2007) use of Toulmin’s (1959) argument structures versus Dimitrova’s (2003) use of Pilkington’s (1999) scheme). Table 2 lists the proposed general terminology for dialogue moves in column 1, with further breakdown in column 2 and examples in column 3. The negotiation moves are designed for practical use in a system, for a range of users, rather than to follow a particular theoretical approach to argumentation. As some of the systems investigated have based their negotiation or discussion on theoretical models, our use of these as a starting point could be said to encompass these approaches.

**Table 2:** Negotiation or discussion moves

Discussion move	Breakdown	Examples
Statement (or explanation)	Beliefs	I believe that my subtraction is quite weak.
	Evidence for beliefs	I have forgotten how to ‘borrow’.
	Reasoning/explanation	This topic (multiplication) is easier than what you were trying before (division).
	Changes made/being made to model	You are trying to reduce my belief from high to medium.
	Assessment criteria	High grammatical accuracy is evidenced by use of a range of structures, with only occasional slips.
Agree (accept)	Beliefs	I agree with your assessment of me (strong).
	Evidence: artefact/result	I agree that my sentences were right.
	Evidence: reasoning	I agree that the activities leading to this value were easier.
	Compromise	I accept the compromise between our beliefs (medium).
Disagree	Beliefs	I disagree that my understanding is strong.
	Evidence: artefact/result	I disagree because my essay was not so good.
	Evidence: reasoning	I disagree because your answers to the last two exercises were nearly all correct, and these were difficult questions.
	Compromise	I do not accept the compromise between our beliefs.
Challenge	Beliefs	I want to change my knowledge of subtraction to medium.
	Evidence: artefact/result	I have now done some more advanced homework.
	Evidence: reasoning	I think that the problem was too easy to be able to distinguish between high and medium competency.
	Validity of data (for purpose)	Problem 18 did not require you to use the skill we are discussing.
Request info	Beliefs	Do you think that the Earth orbits the Sun?
	Evidence: artefact/result	What is the evidence for your belief that my verb declensions are not good?
	Evidence: reasoning	Although your last answers were good, the examples were available. Without the examples, you found some of the questions tricky.
	Assessment criteria	What do I need to demonstrate to advance to the next stage of this topic?
Move on	Interaction to gain more data	As we disagree, let’s look at multiplication of small numbers again.
	Switch to a topic/concept that is not affected by the disagreement in the learner model	Let’s look at Prolog lists for a while.

Statements of beliefs from both parties (student and system; student and teacher; teacher and system; and perhaps student and peer) are necessary to define the respective viewpoints of each partner, before any negotiation can take place. This negotiation will usually require some kind of justification from one or both parties, for their beliefs about the student’s skills. This is in the form of evidence. In the case where students are discussing their learner model with other people, the actual artefact may be very useful as an evidence source. If the artefact is from an online exercise, that might also be helpful in user-system negotiation (with student or teacher as the user). Results from quizzes can be relevant to both user-system and user-user discussion. Evidence can also be in the form of an update on learning, such as when a learner has completed some homework, or simply a statement that they have forgotten something they had previously known. The latter may be given high weighting if the learner’s judgement in the specific learning context is considered likely to reflect their true state (e.g. older learners at school level will often be sufficiently mature to judge this). In the case of completing additional work, the actual artefact may not be interpretable by the system, but the learner’s judgement of this might be considered valid. While this could be modelled at an individual level based on previous negotiation interactions, in practice it may more likely be determined for the specific context of use (e.g. instructors could decide the relative weighting of their students’ claims compared to other sources of data). Explanations or the reasoning for belief representations will also need to be provided for negotiation to proceed. For example, different types of activity may have different levels of granularity in the results—one activity or system could provide data for a concept that indicates ‘high’, ‘medium’ or ‘low’ as an average outcome, another might give a percentage value, and yet another might have performed its own learner modelling, using complex algorithms. If a teacher sets the weightings from these various contributors of data to the learner model, system explanations of this can be very useful for students, in helping them understand why representations may differ from their expectations. Likewise, student explanations of how they see the relative influence of different activities in the model can be useful for the system (or teacher) to understand their viewpoints about their skills. With the variety of learning data now available, we have to accept that there is greater room for imprecision in the learner model. Assessment criteria are important, so that the learner knows what he or she is being assessed against. It is only with this knowledge that they can fully understand whether they have achieved the goal.

For the users and the system (or users with other users) to negotiate the content of the learner model, there must be a way to agree and disagree with the other party’s viewpoint or argument. At the simplest level, this is to (dis)agree with the beliefs. However, the user or the system might disagree over the evidence. This may be the actual artefact produced, which is or is not included in the learner model value, or the reasoning used to calculate the model value (which may be a simpler weighted algorithm or more complex artificial intelligence-based reasoning technique). The understandability of the reasoning process needs to be considered at the time of designing a negotiated learner model in terms of the complexity required and the extent of the reasoning process that can be usefully externalised to the learner. Arguments relating to artefacts or results as evidence are similar to the above. Agreeing or disagreeing with the reasoning for model data can involve issues such as the relative difficulty of tasks and the validity of the evidence for the purpose for which it is being used (e.g. an activity that returns the value ‘high’ may be too course-grained—it may be that the learner is very close to the lower boundary of the category, and when this combined with a slightly lower percentage score from another activity, they find the resulting visual representation to be lower than they expected (even though it is valid given any weightings applied, which may be hidden from the learner). Compromise may be offered and accepted (or refused ‘disagree’) if the user and system do not agree but are each willing to bring their belief value towards that of the other.

Challenges can come from either partner and are likely to occur following a ‘disagree’ move. Challenging in general reflects the next step of disagreement, with processes for challenging beliefs, evidence and purpose similar to the above descriptions.

Likewise, information about beliefs and evidence can be requested. In addition, the assessment criteria may be requested during negotiation. This request is likely to come from the learner, to enable them to better understand what they need to demonstrate to fulfil the criteria. For example, this can be used together with discussion of the validity of specific data for a certain piece of evidence, or challenges to the system’s reasoning. However, the system may also use the assessment criteria as an explanation of its beliefs.

There may be cases where the user and the system do not come to an agreement over a learner model representation. The system (or user) may proceed by trying to gain further data, or may simply move on, especially if there is an area that can be reasonably tackled that does not require either entry for the learner model representation to be certain.

Thus, negotiation of the learner model can be designed very flexibly. In our context of multiple data sources contributing to the learner model, with use by learners and teachers (and possibly peers and parents), we need a generally understandable approach to learner model negotiation. Learners at a range of levels must be able to understand the visualisations and the negotiation options, as well as be able to use the negotiation interface (e.g. conceptual graph and dialogue games for university students (Dimitrova 2003), and smiley faces and free text input interacting with a chatbot with children (Kerly and Bull 2008)). Teachers may also need or want to interact about their students’ learner models (e.g. teacher inspection of learner explanations that are not interpreted by the system—the teacher may wish to contribute new evidence following discussion with a learner, or after seeing the learner’s explanations submitted or recorded in a system (Bull and Vatrapu 2012; Zapata-Rivera and Greer 2004). Although there are, as yet, no examples, a system might usefully involve the teacher in negotiation about evidence, weightings and reasons for evidence, to then be able to communicate with the learner about this. Furthermore, since OLMs may visualise a group model (e.g. Chen et al. 2007; Paiva 1997; Rueda et al. 2006; Tongchai and Brna 2005; Upton and Kay 2009), some kind of negotiation involving group members may be usefully supported.

### An illustration for negotiating learner models with multiple sources of data

**Fig. 2:**
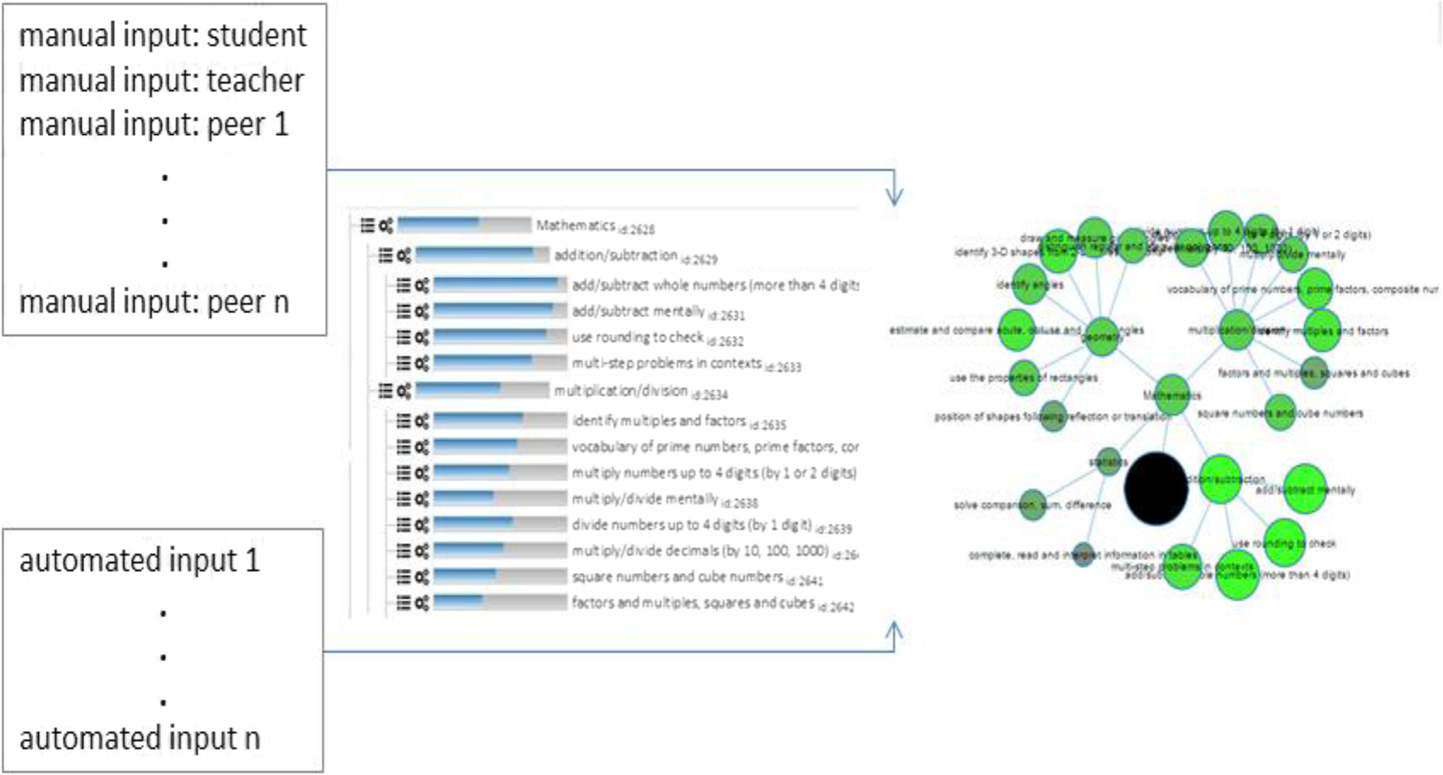
An OLM with multiple sources of data

To conclude this section, we provide an example to illustrate the potential for negotiated learner models in cases where the learner model is constructed from multiple activities, tasks and/or systems. A non-negotiated learner model provides the foundations for this. Figure 2 shows, as an example, the Next-TELL OLM skill meters and competency network in a mathematics course (from Fig. 1), together with example sources of data from human input and from external tools through its API (see Bull et al. 2013a). The data sources comprise:

- Manual—student (self-assessment)
- Manual—teacher (teacher assessment)
- Manual—student’s peers (peer assessments)
- Automated assessment data from multiple other learning environments or activities

**Fig. 3:**
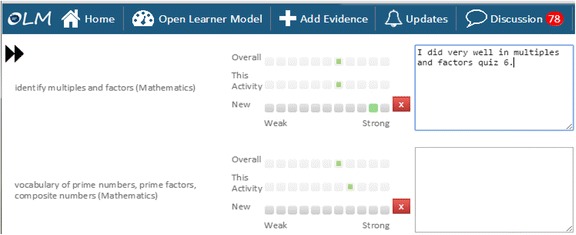
Example of a self-assessment in the Next-TELL OLM

These data sources are combined using a weighted algorithm (with most recent data more heavily weighted), where the weightings for different data can be changed by the teacher, if desired. The interface for adding manual data is shown in Fig. 3. The left-hand side of the screen lists the competencies that are associated with the activity or data source about which the user is entering data. These associations are set up by the teacher, in advance of the OLM being used (see Johnson et al. 2013c). The numerical values that contribute data to each competency in the learner modelling algorithm are input through radio buttons, while additional text can be optionally provided to explain the numerical values (i.e. feedback or guidance from teachers and peers or explanations of self-assessments by students for their teachers). These text descriptions are not interpreted by the system; they are for use solely by the students and teachers.

In addition to tasks away from the computer, a student may be using a range of online activities and standalone systems (as indicated at the bottom left of Fig. 2), which may or may not allow the results or data to be transferred to the OLM. As stated above, in the former case, the assessment data is transferred to the OLM through its API. Developers may most easily allow this by providing a button for learners and/or teachers to transfer the last or current session’s learning data from learning applications to the OLM. In cases where software does not permit data to be automatically transferred, users can provide the information about the outcomes of their use of other systems in the same way that they perform self- and peer assessments (as in Fig. 3). Thus, the Next-TELL OLM already keeps track of the evidence for each source of data (from manual and automated methods). The Next-TELL OLM is currently being extended for negotiation in the LEA’s Box project^1^, where the Tin Can API^2^ (or xAPI) will be used as a basis to connect the OLM to the LEA’s Box Portal and other applications, while still allowing manual entry of data for cases in which the API is not used.

As indicated above, the teacher can adapt the default weightings of activities, to take into account the validity of the data. For example, one external system may perform complex reasoning based on prerequisite relationships, whereas another system may use simpler weighted algorithms, and yet another may produce simple scores. The teacher may therefore decide that systems that use more complex reasoning to compile their results should have higher weighting in the learner model. Similarly, he or she may decide to give higher (or lower) weightings to self or peer assessments, as opposed to other activities. This possibility for teacher intervention in terms of weightings is important, as is the overarching OLM’s need to be able to communicate such information to learners, both to understand what the model is showing (competencies rather than scores) and for negotiation of it.

Returning to our question in the “Terminology and negotiation moves” section of how to initiate learner model negotiation, an additional icon could be placed to the left of the skill meters for learners (or others) to click on (the current icons allow users to see the list of evidence for the learner model data and the calculations that make up the learner model, including the weightings). Alternatively, the labels could be hyperlinks. The discussion might then take place in another area of the screen, or in another window. System initiation of negotiation can be flagged by some kind of notification mechanism or by opening the screen area or additional window and commencing the interaction. The former would allow students to have control over whether they participate in discussion, the latter would more likely favour system control. While this may appear to contradict the interaction symmetry of negotiated learner modelling, the learner could still disregard the system’s new screen area, or the system could still (potentially) ignore a student’s challenge (e.g. if a learner appears to be trying to involve the system in the discussion as a means to avoid another task). In this case, however, the system would likely have mechanisms to guide the learner back towards what they were doing. A short example might run as follows, with the discussion occurring based on any of the methods described in the “Approaches to negotiating and discussing learner models” section, or other approaches. For template text approaches, possible variables that may be selected are shown in square brackets. In this example, the discussion is initiated by the system after recognising the gap between the learner’s self-assessment for division (as input, for example, through the interface shown in Fig. 3) and its own inferences based on online exercises and possibly teacher-provided data. Quotation marks indicate the possibility of free text from the teacher to the student, to further explain the system’s comment. This would not normally be interpreted by the system—unless it is able to analyse dialogue as in, for example, the chatbot-based negotiated learner model (Kerly and Bull 2008).

- SYSTEM (statement-belief): I think your [division] level is [medium].
- LEARNER (challenge-belief): I think my [division] level is [high].
- SYSTEM (request information-evidence): Why do you think your [division] level is [high]?
- LEARNER (statement-evidence): I did [very well] in [Multiplication Quiz 6].
- SYSTEM (statement-evidence): [Multiplication Quiz 6] is for [multiplication].
- SYSTEM (statement-changes): You are trying to change the level for [division].
- LEARNER (challenge-assessment criteria): You need [multiplication] to do [division].
- SYSTEM (disagree-evidence): Your level for [division] is also made up from [Division Quiz 3], [OLMlets_course] and [teacher assessment].
- LEARNER (agree-evidence): I [agree].
- TEACHER (statement-evidence): “A [good] choice. [Multiplication Quiz 6] and [OLMlets] did not help you to learn how to [multiply for division]”.

Other arguments might also have been made, for example, that the other activities undertaken by the learner (Division Quiz 3, OLMlets exercise and a teacher assessment of an artefact) were more recent, and so had higher weighting; that the Multiplication Quiz 6 and/or OLMlets system (Bull et al. 2010) exercises were relatively easy, and that the outcome of other exercises was not as high as the learner claims. This requires that the system keep track of data from different sources, with reference to the competencies to which these sources contribute data. In this case, in Fig. 3 we see that the learner is trying to increase the competency level for the overall competency of division. The numerical equivalent given through the radio buttons is 0.9, higher than the existing value of 0.6 (also shown in Fig. 3). The system identifies this difference in assessments as too great to maintain in the learner model and so initiates negotiation, converting the numerical value of 0.6 to ‘medium’, for discussion. The learner argues that their performance on Multiplication Quiz 6 was high, selecting this quiz from the activities/data sources available and selecting the value ‘high’ as support for their argument. The system accepts that Multiplication Quiz 6 has a high value, but from the teacher’s pre-association of activities/data sources to competencies (see Johnson et al. 2013c), it identifies that this quiz did not contribute to the division competencies. Following the learner’s unaccepted argument in support of their standpoint, the OLM system indicates other activities that yielded a lower value (Division Quiz 3, OLMlets exercise and a teacher assessment). The learner accepts this statement, agreeing with the system’s assessment. The teacher subsequently offers encouragement, acknowledging the learner’s acceptance of the system’s viewpoint and reaffirming the system’s point.

**Fig. 4:**
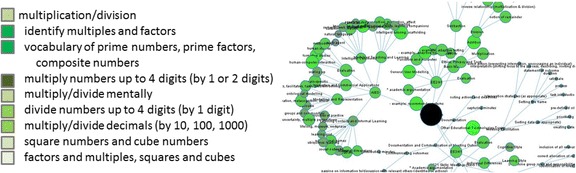
A negotiation initiation example for use alongside a competency network and other expand/collapse or drill-down visualisations

When learner model visualisations are complex, such as the Next-TELL and LEA’s Box OLM competency networks in Fig. 4, another way to initiate negotiation than that proposed above will be required. The size and brightness of the nodes in Fig. 4 represent the learner’s competency level, and nodes can be expanded and contracted to show and hide sub-competencies. In the skill meter example, even when a large set of skill meters is under consideration and scrolling is required, the negotiation can still be initiated in a relatively straightforward way. However, with the competency network example, even if nodes are contracted, it is difficult to include a negotiation ‘initiation point’ directly in the visualisation—clicking on a node will expand or contract it, to help the user to focus on the whole or specific areas, and so this cannot also be used to initiate negotiation. For viewing the evidence and learner model calculations in the Next-TELL OLM, the icons for accessing this information in the visualisations that do not so readily lend themselves to labelling are shown separately at the top right of the screen (shown for the word cloud in Fig. 1). A similar approach could be taken for user-initiation of negotiation in these types of visualisation. Alternatively, a solution such as Prolog-Tutor’s (Tchetagni et al. 2007) left-hand pane listing the skills preceded by coloured squares, where the colour indicates skill level, could be used. An example is given in Fig. 4.

Depending on the specific requirements, the discussion may proceed in another area of the screen, or it may replace the competency network or other visualisation. For example, simpler or small networks may be sufficient in list form for the purpose of negotiation initiation, but if the number of competencies and sub-competencies is larger, it may become more difficult to discuss them if the network is not visible—particularly if the evidence relates to relationships between nodes. In Fig. 4, it is easy to see the lower competencies for the area on the right of the network, by their small size. It may be that this is a new area, and so this difference is expected—but should any small nodes appear amongst a group of larger nodes, this may be a useful trigger for the learner to initiate discussion, or a point to focus on, if the system initiates discussion of this.

**Fig. 5:**
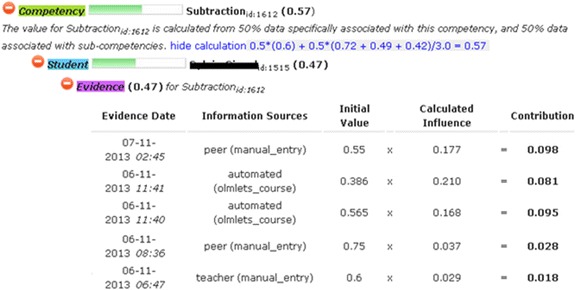
Showing learner model evidence

We finally consider the visualisation of evidence. In the Next-TELL OLM, it is possible to view the evidence list as in Fig. 5 (Johnson et al. 2013a). There is a timestamp, an indication of the source of the learner model data (here: peer assessment, data from the OLMlets system, teacher assessment), the value of the piece of evidence, its influence on the model (weighting), and the resulting contribution of that evidence to the learner model data. The evidence list is given separately for each competency and sub-competency, so learners and teachers can review exactly which activities or data sources contributed the data, and the relative weighting of each piece of data for each competency in the learner model. Thus, each node in Fig. 4, or the corresponding visualisations in Fig. 1, can be explored to identify the underlying data. Therefore, while the Next-TELL OLM is not negotiated, the underlying data is held, providing for a relatively lightweight approach to learner model negotiation (lightweight in the sense that assessments or modelling of specific concepts in external systems cannot be directly accessed—it is the outcome that is transferred to the Next-TELL OLM). The LEA’s Box extension of the Next-TELL OLM is taking this evidence as a starting point for negotiation of the LEA’s Box OLM, initially focusing on allowing students to correct or discuss what they perceive to be inaccurate peer assessments (Bull and Al-Shanfari 2015). Other visualisations may also be used, such as the interactive visual representations that show links between concepts in STyLE-OLM (Dimitrova 2003) and xOLM (Van Labeke et al. 2007).

## Summary and conclusions

This paper has highlighted the growing use of a range of tools and technologies in learning, and the corresponding increasing need for bringing the resulting data together, and keeping it up to date. The field of learning analytics has recently been addressing these issues, with classroom visualisations often aimed at teachers, providing interaction, activity or behavioural data. However, through learner modelling, open learner models display data that has already been interpreted, making it meaningful for the user—not only the teacher but also (and especially) the learner. Combining data that comes from multiple sources, as in the Next-TELL and LEA’s Box OLMs, allows learners to see how their competencies have developed with reference to the sources of data that make up the representations of their competencies.

We have recognised that it may be less likely that this type of OLM will be as fine-grained and accurate as an OLM in a single intelligent tutoring system, or an independent OLM for a specific domain. The external systems may not pass on all the data that would be required for this. Nevertheless, it is still useful to combine data from these sources with other ‘straightforward’ data or scores, given that all activities will contribute in some way to the learner’s knowledge or competencies.

As a means to help maintain the accuracy of OLMs which comprise data from a range of sources, a negotiated learner modelling approach has been proposed. This has been illustrated with an example, showing the need for a system and user to be able to discuss the learner model using options such as statements, agreement/disagreement, challenges and requests for information; and being able to refer to user and system beliefs about the learner’s understanding, skill level and competencies, and the evidence that led to these beliefs. A ‘move on’ option is also desirable in case a disagreement cannot be resolved, or if a learner appears to be trying to game the system. Several approaches have been presented, as used in previous negotiated learner models or learner models that allow discussion of the model content, so the short example given in the “An illustration for negotiating learner models with multiple sources of data” section is intended purely as an illustration. Furthermore, while negotiated learner models have been the primary objective in this paper, there will be cases where the non-symmetrical use of learner model discussion will apply—e.g. if a teacher does not wish the learner to be able to influence the content of their learner model without the teacher’s approval, or if the context of learning demands that the learner should be in control of their learning. We focused more on negotiation as it is the more complex option—designing a learner model for another type of discussion (e.g. persuading the system that the learner’s viewpoint on their knowledge is accurate) can be easily based on a more one-sided interpretation of the fully negotiated approach.

Alongside the issue of accuracy of the learner model is its ability to encourage metacognitive behaviours (Bull and Kay 2013). To some extent this is quite achievable with an OLM that is inspectable only (i.e. without discussion), as the very presentation of representations of a learner’s understanding may be sufficient to prompt reflection, self-assessment, planning, etc. However, discussion of the learner model further focuses the learner onto metacognitive activities, since they have to be able to justify their beliefs if they want the system to change the underlying representation. They will come to recognise the kinds of activity that best help them learn, as well as becoming more aware of the learning process itself.

Current work is adapting the Next-TELL OLM in the context of the LEA’s Box project, with a negotiation component based on the example above. Its specific use at school level, where teachers are also heavily involved, may result in teachers preferring to take control, or for the system to have control. This is one area that will be investigated—how do teachers feel about learner model discussion in their classrooms? Different adaptations might be required or expected at higher education level, or other lifelong learning contexts, and it is hoped that the examples provided here will be followed up by others, in their specific settings.

## Footnotes

1
www.leas-box.eu

2 tincanapi.com
